# A thyroid tumor extending to the parapharyngeal space

**DOI:** 10.1186/1472-6815-6-3

**Published:** 2006-03-01

**Authors:** Fikret Cetik, Demet Yazici, Aysun Uguz

**Affiliations:** 1 Depatment of Otolaryngology, Cukurova University, Adana, Turkey; 2 Department of Pathology, Cukurova University, Adana, Turkey

## Abstract

**Background:**

The metastasis of papillary thyroid carcinoma to the parapharyngeal space is rare and discussed in the English literature before. Encountering a parapharyngeal mass with cystic appearance on imaging, one should rule out thyroid malignancy as differential diagnosis.

**Case presentation:**

The case presented here is a 22-year-old woman who was referred to our clinic with complaints of painless neck mass, dysphagia and hoarseness for two years. After radiologic and pathological examination, the mass thought to be relevant with the thyroid gland. Peroperatively, the tumor was found to originate from the superior pole of the right thyroid gland, with a narrow stalk, and extended following the neurovascular bundle to the lower part of the parapharyngeal space. The bulk was removed via transservical approach with total thyroidectomy.

**Conclusion:**

The occurrence of the follicular variant of papillary thyroid carcinoma in the parapharyngeal space is extremely rare. The management of this rare case was discussed with the review of literature.

## Background

The parapharyngeal space, an inverted pyramid-shaped region, extends from the skull base to the greater cornu of the hyoid bone [1]. Tumors of this space are rare, accounting for 0, 5 % of head and neck neoplasms [2]. Only 20% of these neoplasms are malignant and 50% of these neoplasms arise from the deep lobe of the parotid gland or minor salivary glands [3].

Although thyroid neoplasms are the most common endocrine tumors in head and neck, thyroid cancer is a relatively uncommon neoplasm [4]. Presentation of the thyroid carcinoma as a neck mass extending into the PPS is very rare [5]. A 22-year-old female who has papillary carcinoma of thyroid extending to the parapharygeal space is presented in this case report.

## Case presentation

A 22-year-old female patient was referred our clinic for evaluation of a painless neck mass, hoarseness and dysphagia. She first noticed the mass in the upper right neck two years earlier without any symptoms. Her medical and family histories were unremarkable.

**Figure 1:**
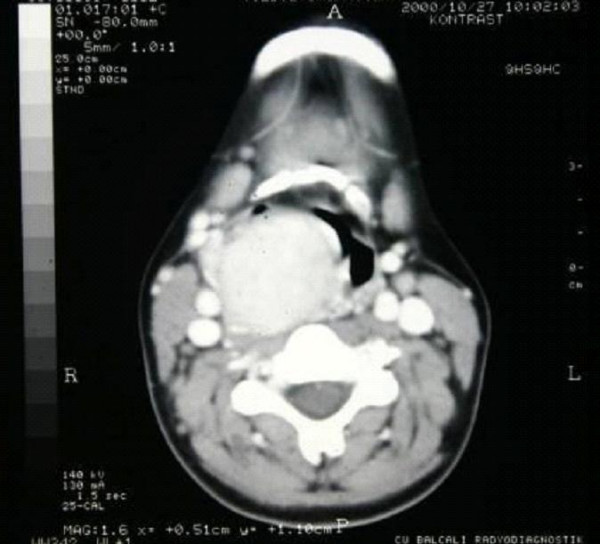
CT scan demonstrated a large, contrast enhancing soft tissue lesion pushing the larynx medially.

**Figure 2:**
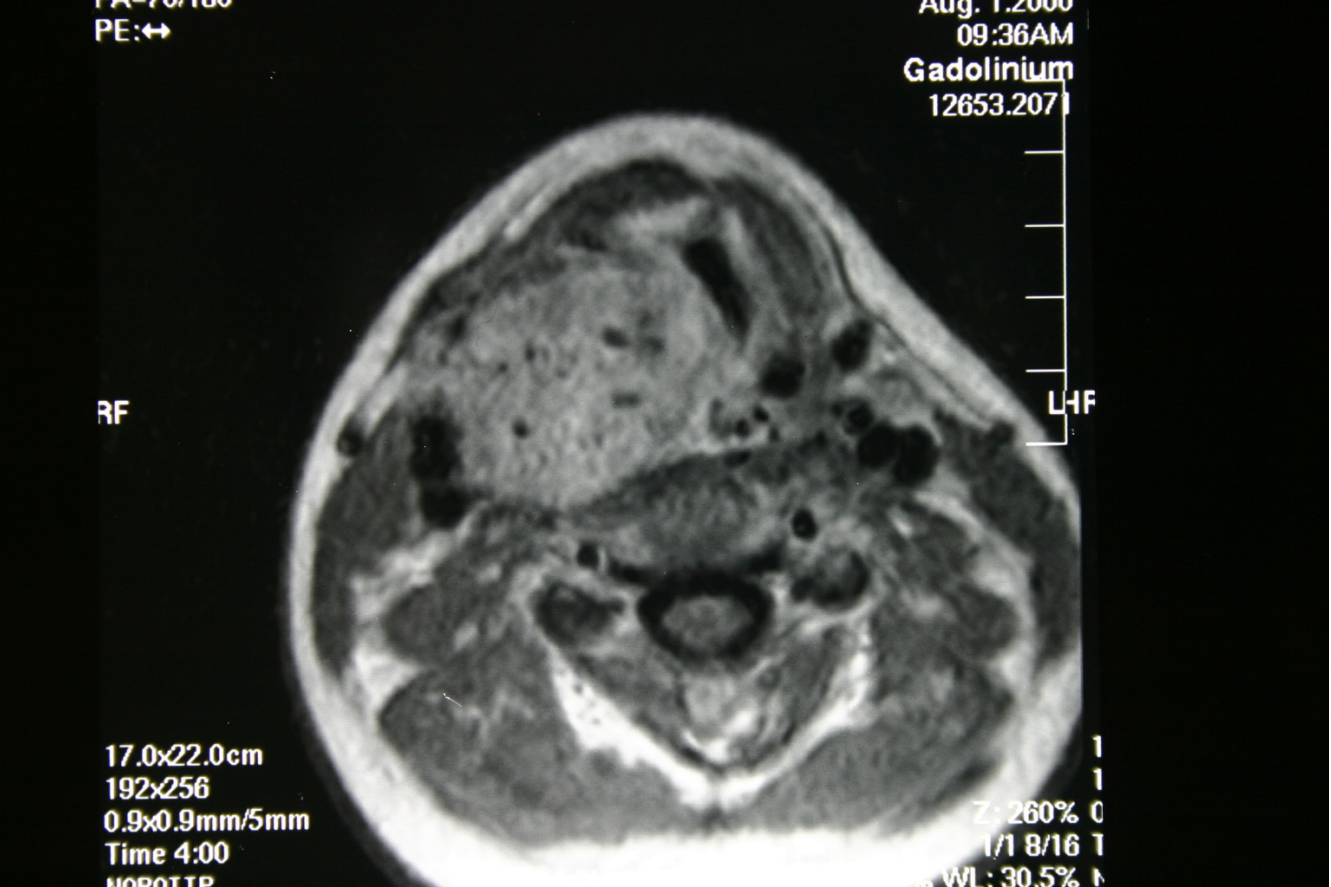
The tumor between the parapharyngeal space and the upper pole of right thyroid lobe was narrowing the airway passage and displacing the carotid artery and the internal jugular vein laterally on MRI.

Physical examination revealed a submucosal mass in the right lateral oropharyngeal wall with medial displacement of the right tonsil. A non-tender, firm, mobile mass measuring 4 × 4 cm. was detected deep to the right sternocleidomastoid muscle below the angle of the mandible. Superior extent of the mass could not be palpated in the neck. The telescopic examination of the larynx demonstrated a right sided, smooth mass narrowing the rima glottis. There were not any cranial nerve deficits other than the paralysis of the right vocal cord. The computed tomographic (CT) scan showed a large, contrast enhancing soft tissue lesion in the parapharyngeal space extending inferiorly to the level of the right thyroid lobe and pushing the larynx medially (Figure 1). The magnetic resonance image (MRI) demonstrated the tumor between the PPS and the upper lobe of right thyroid lobe, narrowing the airway passage and lateral displacement of the carotid artery and the internal jugular vein (Figure 2). The ultrasonographic evaluation of neck demonstrated a 4 × 4, 5 cm mass adjacent to the superior part of the right thyroid gland with rich vascularity.

**Figure 3:**
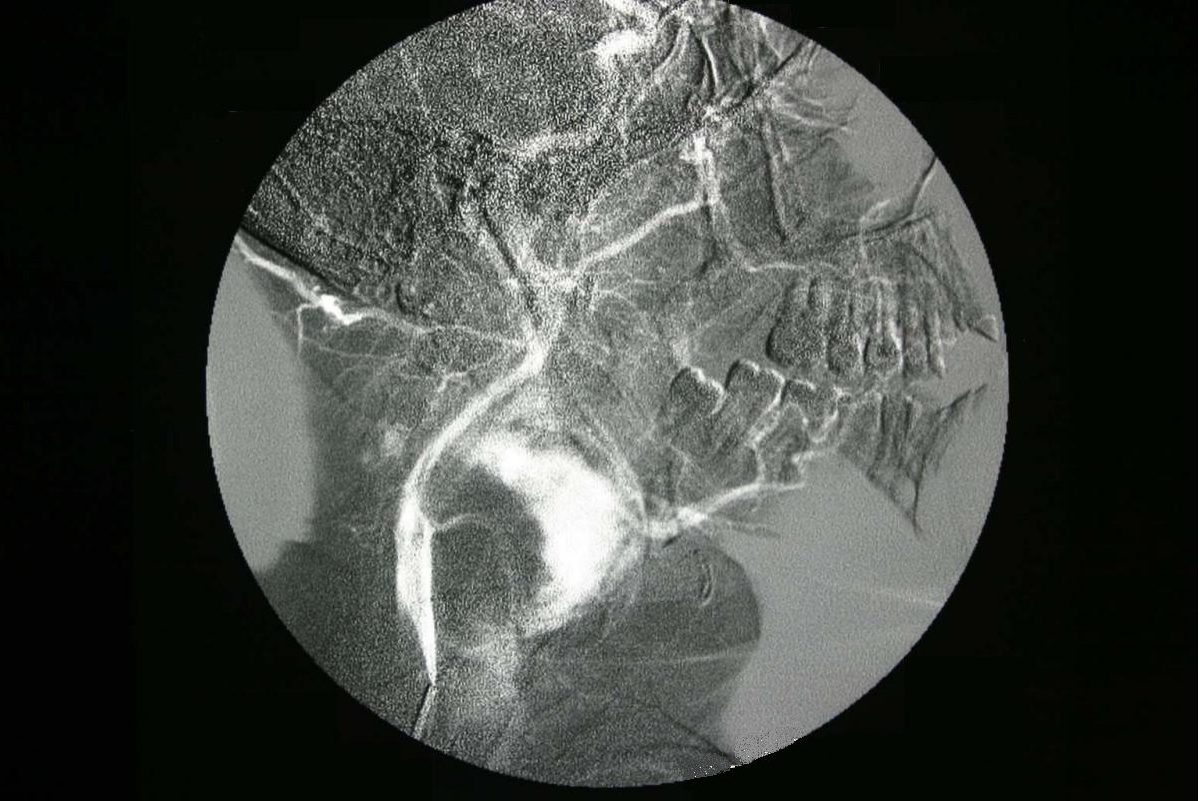
The rich vascularity of the tumor was demonstrated on angiography. Lateral and anteroposterior view.

**Figure 4:**
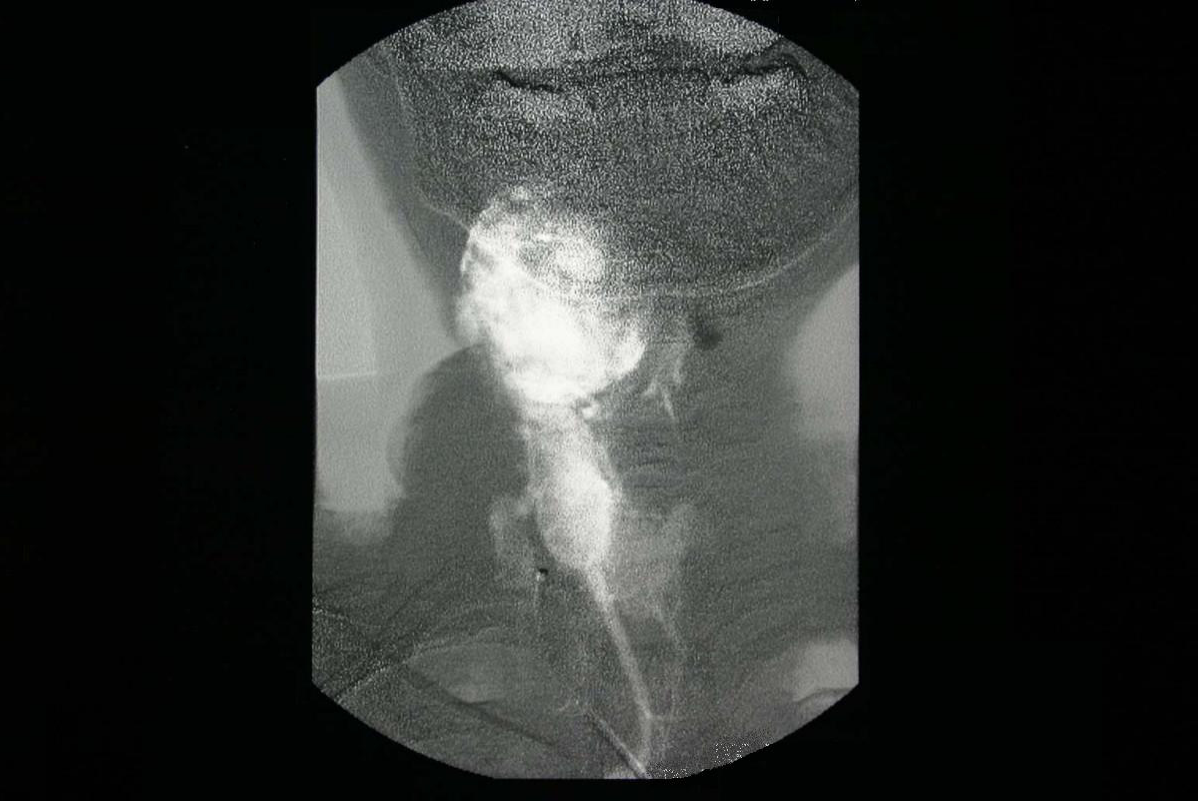
The rich vascularity of the tumor was demonstrated on angiography. Lateral and anteroposterior view.

**Figure 5:**
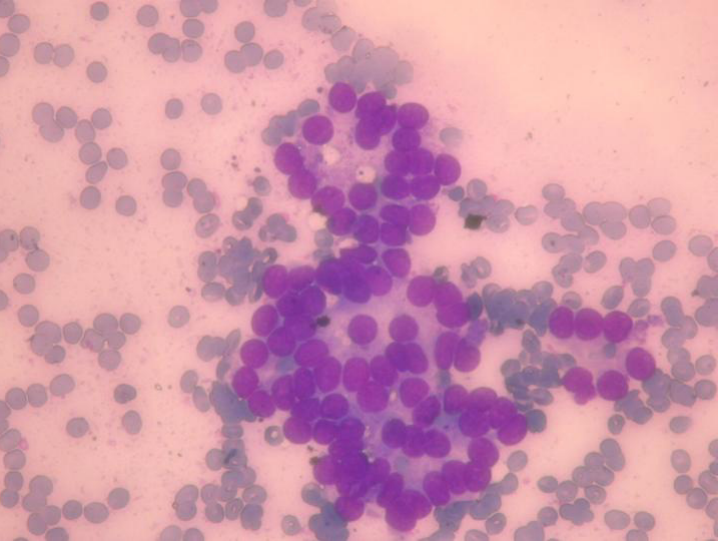
Fine needle aspiration smears of the mass diagnosed as "solid cellular nodule, microfollicular lesion". (May Grunwald Giemsa × 400)

Scintigraphic examination of the thyroid gland revealed homogenous distribution of the activity in the gland but no activity was noticed in the mass adjacent to the gland. Angiography and doppler usg findings suggested that the tumor had rich vascularity and the main blood supply was from the superior thyroid artery (Figure 3, 4). The fine needle aspiration of the mass revealed the cellular aspirates with no colloid, and aspirates which included some microfollicular aggregates. However, no papillary folds, psammoma bodies, or nuclear grooves were detected (Figure 5). It was reported as "solid cellular nodule, microfollicular lesion".

Cervical approach was preferred to remove the mass because it was thought to originate from the right thyroid gland. Under general anesthesia, following the skin incision, starting from the greater cornu of the hyoid bone and extending downwards vertically to the thyroid gland, the tumor with enlarged venous structures and measuring as 4,5 × 4,5 cm was identified. Blunt dissection easily detached the tumor from the pharyngeal constrictor muscles, prevertebral fascia and the skull base. The tumor was found to originate from the superior pole of the right thyroid gland, via a narrow stalk, and extended following the neurovascular bundle to the lower part of the parapharyngeal space. Following the removal of the mass, frozen section was performed, and follicular neoplasm that has suspicion of malignancy was reported. As the frozen artefact of the nuclei of the tumor cells, the exact diagnosis was postponed to the paraffin embedded tissue section. The cervical incision lengthened to gain access to visualize the thyroid gland. Total thyroidectomy was performed with the preservation of the both of the reccurent laryngeal nerves. Careful examination of the median compartment surrounding the thyroid gland did not reveal any lymphadenopathy.

**Figure 6:**
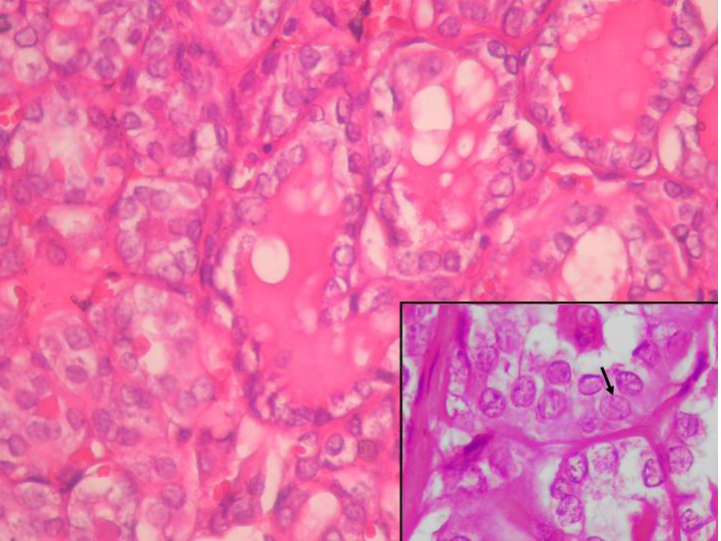
Histologic section of the lesion. Follicular epithelium cells have ground glass nuclei. Arrow was indicated the characteristic nuclear groove of the papillary carcinoma. (Hematoksilen- Eosin × 400).

**Figure 7:**
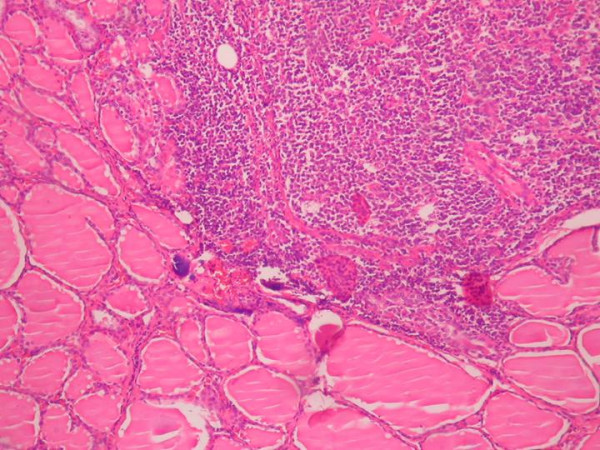
Lymphocytic thyroiditis was observed in the rest of the thyroid tissue. (Hematoksilen-Eosin × 100)

On histologic examination, follicular variant of papillary carcinoma was diagnosed. The tumor had totally follicular architecture and the nuclei of the follicle epithelium were characteristic nuclei of the papillary carcinoma. The cells contained finely dispersed chromatin, which imparted empty appearance, giving rise to the designation glass nuclei (Figure 6). The rest of the thyroid revealed lymhocytic thyroditis (Figure 7). The patient did well postoperatively. There was no residual thyroid in postoperative I-131 scanning. She is free of disease for a follow up of five years.

## Conclusion

The parapharyngeal space is a potential space representing an inverted pyramidal shape with its base at skull base and apex at the greater cornu of hyoid bone. This space contains loose connective tissue, lymphatic vessels, lymph nodes and contents of the carotid sheath and is medially bound by the buccopharyngeal fascia covering the pharyngobasilar plane and the superior pharyngeal constrictor muscle and laterally by the ramus of the mandible and the medial pterygoid muscle.

This potential space is compartmentalized as prestyloid and poststyloid regions by thick fascial layers extending from the styloid process to the tensor veli palatine muscle, called as the tensor-vascular-styloid fascia, composed of the tensor veli palatine muscle itself, its fascia, the stylopharyngeal muscle and the styloglossus muscle. And, its these fascial layers that direct the tumor growth. [3]

As the prestyloid compartment of this potential space consists of the retromandibular portion of the parotid gland, the lymph nodes of the parotid gland and adipose tissue, the poststyloid region consists of internal carotid artery, internal jugular vein, the 9^th^, 10^th^, 11^th ^and 12^th ^cranial nerves, the sympathic chain and the lymph nodes of the oral cavity, oropharynx, paranasal sinuses and the thyroid gland. While the most common lesion of the prestyloid space is the salivary gland neoplasms, especially pleomorphic adenoma of the parotid gland, the most common lesion of the poststyloid region is neurogenic lesions such as schwannomas and neurofibromas.

Primary tumors (benign or malignant), metastatic lymph nodes, lymph node involvement by lymphoproliferative diseases and tumors arising from adjacent sites that secondarily extend into the parapharyngeal space are the four different types of neoplastic lesions of the parapharyngeal space[2].

The PPS tumors usually present as asymptomatic neck or parapharyngeal masses and they often discovered during routine physical examination. These tumors can also present with dysphia, dyspnea, obstructive sleep apnea syndrome, cranial nerve deficits, Horner syndrome (ptosis, miosis, anhydrosis), pain, hoarseness, dysarthria and trismus. Clinical detection of early PPS lesions is difficult since small tumors cause few symptoms. The tumors must reach to a size of at least 2,5 to 3,0 cm before a mass can be detected clinically [7]. Also, when the PPS tumors cause a subtle fullness in the tonsillar region or in the soft palate they can be misdiagnosed as infections or tonsil tumors. In the presented case the neck mass was 4 × 4 cm in diameter, large enough to be noticed in the neck and in the oral cavity.

Initial evaluation of PPS masses should include a complete head and neck examination. Because the PPS lies deep to the muscle of mastication, the mandible, and the parotid glands, clinical examination remains difficult to assess accurately tumor presence and size. But a mass of considerable size -at least 3 cm- will cause a visible bulge or palpable abnormality of the lateral pharyngeal wall or external neck [8]. For the evaluation of the parapharyngeal mass, CT scanning with contrast medium, MRI study with gadolinium, anjiography or MRI anjiography and laboratory studies for urinary vaniyll mandelic acid and metanephrine levels are useful diagnostic procedures and can give appropriate diagnosis up to 95% of the patients without tissue biopsy[1,6]. And, for the first step diagnose, fine needle aspiration can be performed transorally, transservically or guided by CT or ultrasound.

Usually, the initial assessment of thyroid cancer is a palpable neck mass, an intrathyroidal tumor or a metastatic regional lymphadenopathy. However in some patients the tumor may be clinically occult and can be recognized at the time of surgery for benign thyroid disease. And, in approximately 5% to 14% of cases, the thyroid gland is clinically normal, and the first sign of disease is a solitary lateral neck mass [9]. Less common presentations such as hoarseness, vocal cord paralysis, isolated cervical adenophaty, parapharyngeal masses, hemoptysis and pulmonary metastases, even in the face of clinically normal thyroid glands, have been reported and do engender diagnostic dilemmas [5].

In this case presentation, although physical examination did not reveal the relation between the mass and the thyroid gland, the radiologic evaluations implicated that the tumor originated from the thyroid lobe and the superior thyroid artery was the main blood supply of this mass. Moreover follicular cells seen in the fine needle aspiration from the mass was highly suspicious for thyroid neoplasm.

As we mentioned above, tumors of the PPS are usually salivary gland in origin or derived from local neurogenous structures within the space [10]. Other less common neoplasms include chordoma, lypoma, lymphoma, chemodectoma, rhabdomyoma, chondrosarcoma, desmoid tumor, dermois, ameloblastoma, amyloid tumor, ectomesenchyoma, fibrosarcoma and plasmocytoma [11,12]. In this case the mass in the PPS was a primary follicular variant of thyroid papillary carcinoma. (The medline search did not reveal any cases with a primary tumor in PPS.) Although metastatic papillary carcinomas in the PPS were reported before, this case is the first as a primary thyroid neoplasm causing PPS mass symptoms, such as neck mass, hoarseness and dysphagia.

Surgery is the main treatment of the parapharyngeal space tumors. The surgent must be demiurge of the parapharyngeal space anatomy and must consider the size, the location of the mass, the relationship of the tumor to great vessels and the malignancy suspicion for determining the surgical approach for removing the mass[1].

The surgical approaches can be classified as the transoral approach, the transcervical approach, the transparotid-transcervical approach, the transcervical-transmandibular approach and the infratemporal approach. [1] The preferred approach for the removal of the tumor of this case presented here was transcervical approach because the surgent thought the mass originated from the thyroid gland, and this approach helped to remove the tumor easily. Moreover the elongation of the incision provided an extra exposure to remove the thyroid gland totally.

In this case report, thyroid tumor with the symptoms of parapharyngeal mass was presented to emphasize the importance of preoperative evaluation and selection of the appropriate approach for removal. Moreover, primary thyroid tumor should always be remembered in the differential diagnosis of parapharyngeal masses.

## Competing interests

The author(s) declare that they have no competing interests.

## Authors' contributions

The authors have equally contributed to this case report.

## Pre-publication history

The pre-publication history for this paper can be accessed here:


