# Correction: A thyroid tumor extending to the parapharyngeal space

**DOI:** 10.1186/1472-6815-7-3

**Published:** 2007-05-21

**Authors:** Fikret Cetik, Demet Yazici, Ramazan Gun, Aysun Uguz

**Affiliations:** 1 Department of Otolaryngology, Cukurova University, Adana, Turkey; 2 Department of Pathology, Cukurova University, Adana, Turkey

Although he was listed as an author in the original submission of an article published in *BMC Ear, Nose and Throat Disorders *by Cetik *et al*. [1], Dr Ramazan Gun was not included in the author list of the published version. The authors agree that Dr Gun meets the criteria for authorship, and should be considered to be an author of Cetik *et al*. 2006.

## Pre-publication history

The pre-publication history for this paper can be accessed here:


